# Dual-Targeted Hyaluronic Acid/Albumin Micelle-Like Nanoparticles for the Vectorization of Doxorubicin

**DOI:** 10.3390/pharmaceutics13030304

**Published:** 2021-02-26

**Authors:** Manuela Curcio, Luis Diaz-Gomez, Giuseppe Cirillo, Fiore Pasquale Nicoletta, Antonella Leggio, Francesca Iemma

**Affiliations:** 1 Department of Pharmacy, Health and Nutritional Sciences, University of Calabria, 87036 Rende (CS), Italy; giuseppe.cirillo@unical.it (G.C.); fiore.nicoletta@unical.it (F.P.N.); antonella.leggio@unical.it (A.L.); francesca.iemma@unical.it (F.I.); 2 Departamento de Farmacología, Farmacia y Tecnología Farmacéutica, I+D Farma Group, Facultad de Farmacia and Health Research Institute of Santiago de Compostela (IDIS), Universidade de Santiago de Compostela, 15782 Santiago de Compostela, Spain; luis.diaz.gomez@usc.es

**Keywords:** hyaluronic acid, human serum albumin, polysaccharide-protein conjugate, micelle-like nanoparticles, redox-responsive, active targeting, cancer therapy

## Abstract

Drug targeting of tumor cells is one of the great challenges in cancer therapy; nanoparticles based on natural polymers represent valuable tools to achieve this aim. The ability to respond to environmental signals from the pathological site (e.g., altered redox potential), together with the specific interaction with membrane receptors overexpressed on cancer cells membrane (e.g., CD44 receptors), represent the main features of actively targeted nanoparticles. In this work, redox-responsive micelle-like nanoparticles were prepared by self-assembling of a hyaluronic acid–human serum albumin conjugate containing cystamine moieties acting as a functional spacer. The conjugation procedure consisted of a reductive amination step of hyaluronic acid followed by condensation with albumin. After self-assembling, nanoparticles with a mean size of 70 nm and able to be destabilized in reducing media were obtained. Doxorubicin-loaded nanoparticles modulated drug release rate in response to different redox conditions. Finally, the viability and uptake experiments on healthy (BALB-3T3) and metastatic cancer (MDA-MB-231) cells proved the potential applicability of the proposed system as a drug vector in cancer therapy.

## 1. Introduction

Non-specific targeting and multi-drug resistance are the main limitations to the success of conventional chemotherapy. In the last decades, to overcome these problems, nanoparticle formulations have been proposed as valuable tools to deliver conventional drugs to the site of interest, modifying their pharmacokinetics and therapeutic response [1]. The suitability of nanoparticles in cancer treatment is related to some evidence. Nanoparticles can efficiently accumulate in the tumor site by virtue of angiogenesis and the lack of lymphatic drainage, a condition known as the enhanced permeation and retention (EPR) effect [2]. In addition, the possibility to modify the physicochemical and surface properties of nanoparticles allows the amount of drug delivered to the target site to be remarkably increased. For this purpose, two different approaches can be followed: (i) conjugation with ligands binding to receptors over-expressed onto the tumor cells [3]; (ii) functionalization with chemical groups able to respond to signals from tumor microenvironments [4,5]. These engineered materials, known as actively-targeted drug delivery systems, have emerged as “magic bullets” able to hit the site of disease, avoiding the side effects to other healthy organs [6]. Small molecules [7] or macromolecular compounds [8] are employed as active targeting ligands, whereas the variation of pH, redox potential, temperature or reactive oxygen species concentration are the most exploited environmental stimuli in a tumor-targeted release [9]. For example, it is well known that the remarkable differences in glutathione (GSH) concentration in cancer cells (2–10 mM), compared to the normal extracellular matrix (2–20 μM), generate a high redox potential [10]. Thus, by inserting in the nanoparticle structure redox-responsive functionalities, i.e., disulfide linkages [11], materials able to selectively destabilize inside cells and release the payload can be achieved.

To date, the wide availability of materials and preparation methods allows for a large number of nanoparticle architectures to be obtained endowed with different targeting ligands and stimuli-responsive chemical groups. Natural polymers, such as polysaccharides and proteins, have emerged as promising base materials of drug delivery platforms in cancer therapy due to their unique physicochemical and biological properties such as non-immunogenicity, biocompatibility, biodegradability, and the presence of several functional groups available for chemical modification [12]. Protein nanoparticles can be prepared under mild conditions without the use of toxic chemicals or organic solvents, allowing to easily control the particle size [13,14]; on the other hand, polysaccharide materials are safe and may confer “stealth” properties to the final nanoparticle system, decreasing the uptake by the mononuclear phagocyte system and enhancing the blood circulation time and the possibility of accumulation in the disease site [15]. These valuable properties have inspired the preparation of protein-polysaccharide conjugates showing superior features not attainable using the individual component [16,17].

Human serum albumin (HSA) is the most abundant protein in the blood and one of the most attractive materials for the development of novel nanomedicines [18,19] due to its high drug-loading ability, self-assembling properties, and long half-life. These properties, among others, like its lack of toxicity and immunogenicity and its preferential uptake in tumor and inflamed tissues, make HSA a promising candidate for drug delivery. Furthermore, the tertiary structure of HSA contains a drug-binding site (subdomain I B) that has a remarkable affinity for doxorubicin, among other antitumor drugs [20,21]. The increased hydrophobicity of HSA after binding between doxorubicin significantly enhances the self-assembly properties of the protein [22]. HSA nanoparticles have shown active tumor targeting via receptor-mediated endocytosis, including GP60 and neonatal Fc receptors that are overexpressed in tumor cells. Moreover, recent studies have reported that HSA nanoparticles undergo aggregation in a tumor pH environment, thus favoring accumulation and retention of HSA-drug complexes in tumor tissues [23].

Another approach to achieve active drug targeting is the incorporation of hyaluronic acid (HA) into nanoparticle systems. HA is a negatively charged, non-sulfated glycosaminoglycan composed of repeating units of d-glucuronic acid and *N*-acetyl-d-glucosamine bound by beta-linkages and is found throughout the body in the extracellular matrix and synovial fluids [24]. HA-based nanoparticles have been extensively explored as delivery devices in cancer therapy by virtue of their ability to specifically bind CD44 receptor overexpressed on various cancer cells [25]. Furthermore, it is possible to endorse HA nanoparticles with redox-responsive properties by cystamine decoration, enhancing the desired targeting properties for tissues exhibiting increased GSH levels, such as tumors [26].

In this work, we aimed to develop a novel drug delivery system with redox-responsive and actively targeting properties for anticancer therapies. Micelle-like nanoparticles were obtained by a simple self-assembling process of a conjugate (HAssHSA) composed of cystamine-modified HA (HAcys) and HSA. We hypothesized that the bioconjugation of HAssHSA and the resulting self-assembling nanoparticles (HNPs) could merge the advantageous features of each component in a nanoparticle system with enhanced targeting properties for CD44-overexpressing cells, redox responsiveness, and high loading ability. The obtained nanoparticles were characterized by dynamic light scattering (DLS) and transmission electron microscopy (TEM), and redox-triggered destabilization assays were performed by measuring the variation of the nanoparticle mean diameter in reducing media. The suitability of HNPs as drug carriers was then evaluated by loading with doxorubicin hydrochloride (DOX), a DNA topoisomerase II inhibitor with a broad-spectrum antineoplastic activity [27]. However, DOX use in clinical practice is often limited by several side effects. In vitro release profiles from DOX-loaded HNPs were investigated, varying the redox potential of the surrounding medium. Finally, cytotoxicity and cellular uptake experiments were performed on healthy and cancer cells to assess the safety the potential suitability of the system in cancer therapy.

## 2. Materials and Methods

### 2.1. Synthesis of HAcys

HAcys was obtained by reductive amination [28,29]. Briefly, after 0.1 g (0.24 mmol disaccharide repeating units) of HA (10 kDa) were dissolved in 4 mL of an H_2_O:DMSO (3:7 *v/v*) mixture, 0.31 g (4.93 mmol) of sodium cyanoborohydride and 1.1 g (4.45 mmol) of cystamine dihydrochloride (cysHCl) were added, and the mixture was stirred for 24 h at room temperature. The resulting solution was purified using dialysis membranes with molecular weight cutoff (MWCO) of 3.5 kDa against water at 20 °C for 72 h, and finally freeze-dried (98% yield). ^1^H-NMR spectra were recorded on a Bruker Avance 300 (Bruker Italy, Milan, Italy) at 25 °C using D_2_O as a solvent.

HA was purchased from Lifecore Biomedical (Chaska, MN, USA). All other chemicals were purchased from Merck/Sigma-Aldrich, Darmstadt, Germany. Dialysis membranes were purchased from Medicell International LTD (London, UK).

### 2.2. Synthesis of HAssHSA Conjugate

HSA (0.074 g) and 1-ethyl-3-(3-dimethylaminopropyl) carbodiimide (EDC) (0.010 g, 0.052 mmol) were dissolved in 4 mL of a phosphate buffer solution (0.01 M, pH 7.4) and left to react for 1 h at room temperature under magnetic stirring. Then, HAcys (0.017 g) dissolved in 2 mL of phosphate buffer (0.01 M, pH 7.4) was added. The mixture was magnetically stirred for 24 h at room temperature, purified by dialysis (MWCO 12–14 kDa) against water at 20 °C for 72 h, and finally freeze-dried (98% yield). ^1^H-NMR spectra were recorded on a Bruker Avance 300 (Bruker Italy, Milan, Italy) at 25 °C using D_2_O as a solvent.

All chemicals were purchased from Merck/Sigma-Aldrich, Darmstadt, Germany. Dialysis membranes were purchased from Medicell International LTD (London, UK).

### 2.3. Preparation of Labeled HAssHSA Conjugate

For the synthesis of the labeled conjugate, fluorescein isothiocyanate (FITC) was covalently linked to the HAssHSA as previously described [30]. HAssHSA (60 mg) was dissolved in 3.0 mL sodium carbonate buffer (100 mM, pH 9.2), and then 0.6 mL FITC solution in sodium carbonate buffer (1.0 mg mL^−1^) was added and allowed to react at RT for 3 h. The obtained FITC-HAssHSA was purified by dialysis (MWCO 12–14 kDa) against 5.0 mM phosphate buffer (pH 3.0) for two days and characterized by UV–Vis spectra. UV–Vis analysis was performed on an Evolution 201 spectrophotometer (Thermo Fisher Scientific, Hillsboro, OR, USA) operating with 1.0 cm quartz cells. All chemicals were purchased from Merck/Sigma-Aldrich, Darmstadt, Germany.

### 2.4. Determination of the Critical Aggregation Concentration (CAC)

The modification of the fluorescence properties of the nonpolar probe pyrene, when located inside a micelle hydrophobic core, was exploited to measure the CAC of HAssHSA in the aqueous phase [31,32]. In detail, 20.0 μL pyrene solution at a concentration of 3.0 × 10^−5^ M in acetone was evaporated in vials. HAssHSA conjugate was dissolved at concentrations ranged from 1.6 × 10^−7^ to 1 mg mL^−1^ in phosphate buffer (0.01 M, pH 7.4) under magnetic stirring, and then 1 mL of each solution was added to the pyrene vials. The contents of the vials were mixed for 12 h, thereby leading to solutions with a pyrene concentration of ca. 6.0 × 10^−7^ M. Then, the intensity ratios (I_3_/I_1_) of the third vibronic band at 385 nm to the first one at 373 nm of the fluorescence emission spectra of pyrene were recorded at 25 °C. Pyrene fluorescence emission spectra (λ_exc_ = 336 nm; λ_em_ = 350–500 nm) were recorded on Hitachi F-2500 spectrometer (Tokyo, Japan). All chemicals were purchased from Merck/Sigma-Aldrich, Darmstadt, Germany.

### 2.5. Preparation and Characterization of Micelle-Like Nanoparticles

Empty nanoparticle HNPs were obtained by dispersing the HAssHSA conjugate (final concentration of 1 mg mL^−1^) in phosphate buffer solution (0.01 M, pH 7.4) under magnetic stirring for 2 h at room temperature. For the cell uptake experiments, FITC-HAssHSA was treated in the same manner, obtaining the fluorescent-labeled FITC-HNPs. The size distribution of HNPs (final concentration 1 mg mL^−1^) was determined using a 90 Plus particle size analyzer DLS equipment (Brookhaven Instruments Corporation, Holtsville, NY, USA) at 25 °C. The autocorrelation function was measured at 90°, and the laser beam operated at 658 nm. The polydispersity index (PDI) was directly obtained from the instrumental data fitting procedures by the inverse Laplace transformation and Contin methods. PDI values ≤ 0.3 indicate homogenous and mono-disperse populations [33]. Morphological analysis of vesicles was carried out using TEM (HRTEM/Tecnai F30 (80 kV) FEI Company, Hillsboro, OR, USA). A drop of the vesicle dispersion was placed on a Cu TEM grid (200 mesh, Plano GmbH, Wetzlar, Germany), and the sample in excess was removed using a piece of filter paper. A drop of 2% (*w/v*) phosphotungstic acid solution was then deposited on the carbon grid for 2 min. Once the excess staining agent was removed with filter paper, the samples were air-dried, and the thin film of stained vesicles was observed.

All chemicals were purchased from Merck/Sigma-Aldrich, Darmstadt, Germany.

### 2.6. Destabilization Experiments

Destabilization experiments of empty nanoparticles in reductive environments were performed by the dialysis method. Briefly, in separate experiments, 4 mL of freshly prepared empty nanoparticles (final concentration 1 mg mL^−1^) were loaded in a dialysis bag (MWCO 12–14 kDa) and dialyzed against 30 mL phosphate buffer (0.01 M, pH 7.4) containing GSH at different concentrations (0 mM and 10 mM) at 37 °C in a beaker with constant stirring. After 24 h, the mean diameter and PDI were measured by DLS. Each analysis was performed in triplicate.

### 2.7. DOX Loading

DOX-loaded nanoparticles (DOX@HNPs) were prepared by dispersing HAssHSA conjugate (final concentration 1 mg mL^−1^) in a 58.8 μM DOX hydrochloride solution in phosphate buffer (0.01 M, pH 7.4) under magnetic stirring for 12 h at room temperature [34]. The dispersion was used as such in the next release experiments. The loading efficiency was confirmed by diluting 1 mL of DOX@HNPs dispersion in 25 mL of methanol in order to disrupt the micelle-like structure [35], followed by the measurement of the fluorescence of the solution (λ_exc_ = 480 nm; λ_em_ = 590 nm).

### 2.8. Release Experiments

Release experiments were carried out by means of a dialysis method under sink conditions. In two different experiments, 2 mL DOX@HNPs dispersion (polymer concentration 1 mg mL^−1^) were loaded in a dialysis bag (MWCO 3.5 kDa) and dialyzed against 10 mL phosphate (0.01 M, pH 7.4) buffer containing GSH at different concentrations (0 mM and 10 mM) at 37 °C in a beaker with constant stirring. At pre-established times, samples (0.5 mL) of release medium were withdrawn, replaced with fresh medium and quantified by a fluorescence spectrometer (λ_exc_ = 480 nm; λ_em_ = 590 nm) using a standard calibration curve of and DOX (2–30 μM) prepared under the same conditions. Experiments were performed in triplicate.

### 2.9. Cytotoxicity Experiments

MDA-MB231 (Cell Biolabs; San Diego, CA, USA) and BALB/3T3 (CCL-163; ATCC, Manassas, VA, USA) cell lines were maintained in DMEM supplemented with 10 *v/v*% FBS and 1% antibiotics (10,000 U/mL penicillin and 10,000 µg/mL streptomycin) in an incubator at 37 °C and 5% CO_2_.

The cytotoxic effects of the drug, empty nanoparticles and drug-loaded systems on cell viability were determined using a Cell Counting Kit 8 (CCK-8; Dojindo Molecular Technologies; Rockville, MD, USA). MDA-MB231 or BALB/3T3 cells were seeded in 96-well plates (1 × 10^4^ cells/well) and incubated overnight. Then, cells were exposed to increasing concentration of the drug (0.001 to 5 µg mL^−1^) or HNPs (0.1 to 2 mg mL^−1^) and further incubated for 24 or 48 h. At each time point, the medium was discarded, cells were washed with PBS, and 100 μL of freshly prepared CCK-8 working solution (90 μL of culture medium and 10 μL of CCK-8 reagent) were added to each well and incubated for 2 h at 37 °C. Finally, absorbance (450 nm) was recorded using a microplate reader (Model 8; Bio-Rad, Philadelphia, PA, USA). Cell viability was determined by comparing the absorbance of each condition with a positive control consisting of cells incubated in the culture medium.

DOX@HNPs cytotoxicity was evaluated in MDA-MB231 and BALB/3T3 cells. For the preparation of DOX-loaded HNPs, 1 mg of HAssHSA was dispersed in 5 mL of freshly prepared DOX solutions (40, 20, 10, and 5 μg mL^−1^) in phosphate buffer under magnetic stirring overnight. Then, 1 mL of each nanoparticle dispersion was added to 3 mL of phosphate buffer in order to obtain a final nanoparticle concentration of 0.025 mg mL^−1^ and DOX concentrations of 5, 2.5, 1.25, and 0.625 μg mL^−1^. MDA-MB231 or BALB/3T3 cells (1 × 10^4^ cells per well) were seeded into 96-well plates and incubated for 24 h as explained before. Then, the medium was removed, and 100 μL of fresh medium and 100 μL of the freshly prepared DOX@HNPs nanoparticle solutions were added to each well and cultured for 24 or 48 h. Phosphate buffer (0.01 M, pH 7.4) was used as the negative control. At each time point, the medium was discarded, cells were washed with PBS and 100 μL of freshly prepared solution (90 μL of culture medium and 10 μL of CCK-8 reagent were added to each well and incubated for 3 h at 37 °C. Absorbance (450 nm) was then recorded using a Model 8 microplate reader (Bio-Rad, USA).

### 2.10. Visualization of CD44-Mediated Cellular Uptake

MDA-MB231 and BALB/3T3 cells were cultured as explained in Section 2.6 and seeded in 8-well glass slides (Lab-Tek II chamber slide; Thermo Scientific, Waltham, MA, USA) at a concentration of 5 × 10^4^ cells/well. After 24 h of incubation, cells were washed with PBS and then, CD44 receptors were blocked by incubating cells with 10 equivalents of free HA (solution in culture medium) for 1 h. Untreated cells were used as positive controls. Subsequently, cells were washed with PBS and treated with FITC-HNPs (0.1 mg mL^−1^) for 24 h. At each time point, cells were washed with PBS, fixed with paraformaldehyde 4% (100 μL per well) for 10 min, washed three times again with PBS and then, incubated with Triton X-100 (0.2% in PBS pH 7.4, 40 μL, 5 min), and washed three times again with PBS. Then, cells were stained with a phalloidin dye (Alexa Fluor 488 phalloidin, Molecular Probes; Eugene, OR, USA) for 20 min following the manufacturer’s protocol and washed again with PBS. Subsequently, samples were mounted using DAPI ProLong gold (Molecular Probes; Eugene, OR, USA), covered with a cover glass, and frozen until observation. Confocal images were recorded using a Leica confocal TCS-SP5 (Leica Microsystems; Wetzlar, Germany). The evaluation of cellular uptake of DOX-loaded HNPs (DOX@HNPs) was also carried out using confocal microscopy. Cells were seeded, and CD44 receptors blocked, as explained before. Following this, cells were treated with culture medium (negative control), a 0.8 mg mL^−1^ DOX solution or DOX@HNPs (containing 0.8 mg mL^−1^ of DOX), and incubated for 12 h. Finally, cells were fixed and stained as described before and observed using a Leica confocal TCS-SP5 (Leica Microsystems; Wetzlar, Germany).

### 2.11. Statistical Analysis

Statistical analyses were analyzed using GraphPad Prism (GraphPad Software, La Jolla, CA, USA). All results were expressed as mean values with standard deviations. Two-way analysis of variance and Tukey’s multiple comparison post-test were used. Differences were considered significant for *p* < 0.05.

## 3. Results and Discussion

### 3.1. Synthesis of HAssHSA Conjugate

**Figure 1:**
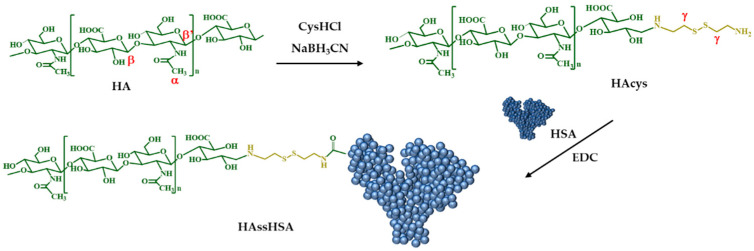
Synthesis of HAcys and HAssHSA conjugate.

The covalent conjugation of biomacromolecules is attracting increasing interest because of the possibility to combine the advantages of each component, minimizing their respective liabilities [36]. HAssHSA conjugate was obtained in a two-step procedure consisting in (i) the synthesis of HAcys by reductive amination, a selective, fast and easy reaction procedure using cysHCl and sodium cyanoborohydride as reactants; and (ii) the conjugation of HAcys with HSA via covalent coupling with EDC (Figure 1).

**Figure 2:**
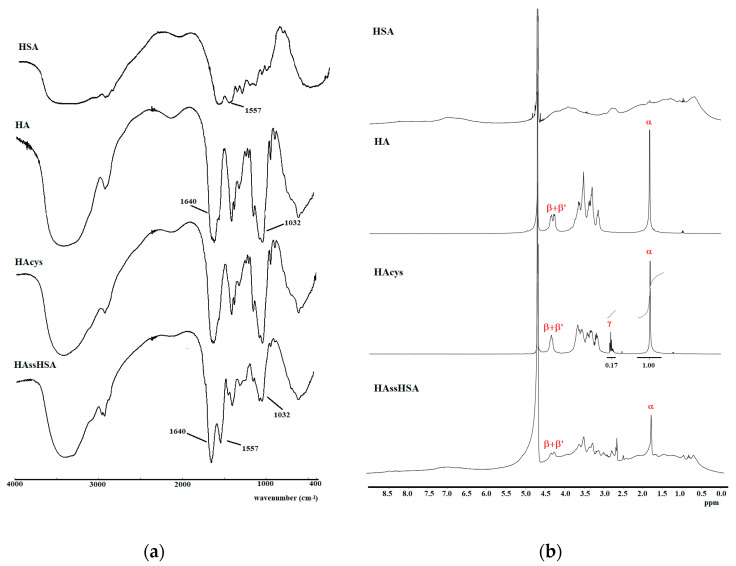
FT-IR (**a**) and ^1^H-NMR (**b**) spectra of HSA, HA, HAcys and HAssHSA.

HAcys and HAssHSA were characterized by FT-IR and ^1^H-NMR analyses. As depicted in Figure 2a, the FT-IR spectrum of HAcys did not show any significative variation compared to native HA because the absorption bands of cystamine were overshadowed by the more intense absorption bands of the polysaccharide. In the ^1^H-NMR spectra (Figure 2b), the signals of the two HA anomeric protons (β and β’) were evident at around 4.30 ppm, while the presence of cystamine residues was quantitatively determined from relative integration of peak (α) at 1.81 ppm corresponding to the three methyl protons of N-acetyl group of HA and the triplet signal (γ) at 2.80 ppm related to the four methylene protons of cystamine. The derivatization degree (DD), expressed as cystamine moieties with respect to HA repeating units, was calculated to be 12.7%.

The FT-IR spectrum of HAssHSA conjugate showed the typical absorption bands of HA (1640 and 1032 cm^−1^), corresponding to the stretching vibrations of amide carbonyl and C–OH, respectively). An absorption band at 1557 cm^−1^ ascribable to the secondary amide band of HSA was also present in the HAssHSA spectrum, confirming the successful polysaccharide-protein conjugation. Furthermore, the conjugation was also evidenced by the presence of observable peaks in the aromatic region (8.50–6.50 ppm) and aliphatic region (0.50–1.80 ppm) of the ^1^H-NMR spectrum of HAssHSA.

**Figure 3:**
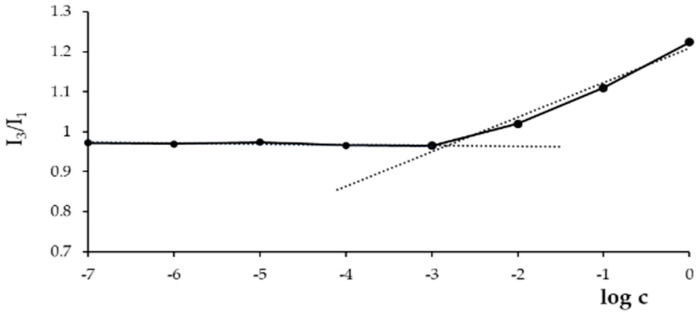
Dependence of pyrene fluorescence spectrum signals on HAssHSA concentration at pH 7.4.

The self-assembling capability of the HAssHSA conjugate was evaluated by measuring its CAC, using pyrene as a fluorescent probe for hydrophobic domain formation. The determination of CAC is pivotal in view of a potential application in vivo, where the system is highly diluted in the systemic circulation: the lower the CAC value, the greater the stability of the conjugate that will be able to organize at low concentrations. For this measurement, the dependence of pyrene fluorescence spectra (I_384_/I_373_ ratio) on the logarithm of conjugate concentration was considered, and the results are depicted in Figure 3.

At low concentrations, the intensity values remained almost unchanged; when the amount of conjugate increased, a sharp change of the intensity was observed, indicating the onset of self-assembly. From the crossover points, a CAC of 1.6 μg mL^−1^ was calculated in accordance with the data reported in the literature for HA-based nanoparticles [37,38]. It is well-known that the amphiphilic properties of HSA, a globular protein with hydrophobic domains in the inner core and hydrophilic domains in the surface layer, enable self-assembling into micellar structures [39,40,41]. In fact, it was recently reported that HA-albumin conjugates could self-assemble into uniform micelle-like structures [42].

### 3.2. Preparation of HNPs and DOX Loading

**Figure 4:**
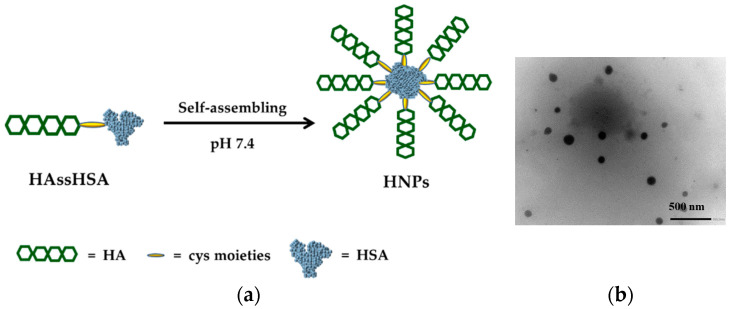
(**a**) Proposed architecture of the vesicle membrane and (**b**) TEM image of negatively stained HNPs (20 K magnification).

HNPs were prepared in a straight-forward procedure by dispersing the HAssHSA conjugate in phosphate buffer solution at pH 7.4, exploiting the amphiphilic character of the system. It was hypothesized that the hydrophobic core is constituted by the hydrophobic domains of HSA, while the hydrophilic HA chains form the shell of the micelle-like nanoparticles [42] (Figure 4a).

DLS analysis showed a unimodal particle size distribution, with a mean hydrodynamic diameter of 70 ± 9 nm and a PDI of 0.2, while TEM micrographs revealed that the HNPs were characterized by spherical shape and a mean diameter value close to that recorded by DLS (Figure 4b). The size of the HNPs described in this work is significantly smaller compared to previously reported HSA nanoparticles prepared by the desolvation method [43,44,45] or self-assembling processes [46,47,48]. Particle size is the result of the combination of different parameters, including physic-chemical properties of the amphiphile, preparation method, and experimental conditions, that strongly affect the rearrangement of the polymer structure during the self-assembling step. HNPs dimensions present an ideal size that is large enough to accumulate in tumor tissues via enhanced permeability and retention (EPR) effect. However, since the particle size is lower than 100 nm, it is expected to achieve extensive tumor penetration [49].

### 3.3. Destabilization Experiments

GSH-responsive behavior of HNPs was assessed by means of destabilization experiments performed in media mimicking the extracellular and intracellular redox environments. In particular, the variation of the dimensional distribution of micelle-like nanoparticles was analyzed by DLS after 24 h incubation in phosphate buffer at physiological pH with or without GSH 10 mM. The mean diameter of HNPs remained almost unchanged in the medium, mimicking the extracellular environment, while in the presence of the tripeptide GSH both the mean diameter and the PDI dramatically increased to 130 nm and 0.54, respectively. This observation confirms that HNPs were responsive to GSH, which can be explained as a consequence of the reduction of disulfide bonds, resulting in the destabilization of the nanoparticle structure [9,50].

### 3.4. DOX-Loading and Release Experiments

A stimuli-responsive drug delivery system is expected to quickly release its payload when targeted tissues or cells are reached while remaining stable in the systemic circulation. In this work, DOX hydrochloride, employed as a model drug, was loaded in the nanoparticles in a one-step procedure, avoiding the need for organic solvents or H_2_O_2_ to solubilize the components or to initiate the molecular assembly of the drug-nanoparticle complexes [43,51,52]. The drug was added at a concentration of 32 mg per g of conjugate during the self-assembling step, exploiting the strong affinity between DOX and HSA [22,53,54], and the resulting DOX@HNPs dispersion was used as such in the release experiments assuming that the total amount of added DOX was absorbed by the nanoparticles. This assumption is supported by literature data reporting the range of DOX:HSA ratio where the dissolved protein can completely absorb the drug from a water solution [46].

Release experiments from DOX@HNPs were performed in media mimicking the physiological conditions (phosphate buffer at pH 7.4) and the intracellular space (GSH 10 mM) [55,56]. A controlled release profile was recorded at pH 7.4 simulating normal physiological conditions: after a slight burst release after the first 30 min (15%), the DOX cumulative percentage in the releasing medium does not exceed 26%, indicating that the DOX@HNPs are stable in the extracellular medium of healthy tissues and only a small amount of drug would be leaked during blood circulation. These results reflect the high-affinity between DOX and HSA in aqueous media at pH values close to physiological conditions. In fact, recent studies have shown that the hydrophilic and hydrophobic interactions between DOX and albumin result in the formation of more stable complexes in HSA than other albumins, such as BSA [21].

**Figure 5:**
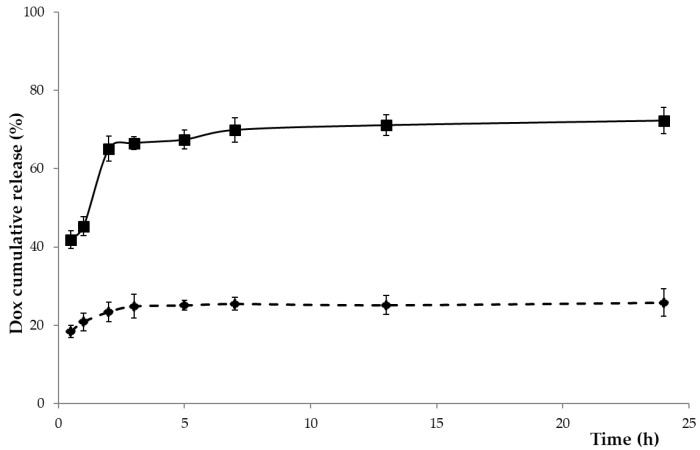
Drug release profiles of doxorubicin hydrochloride (DOX) from DOX@HNPs in phosphate buffer at pH 7.4 without (dashed line) and with glutathione (GSH) 10 mM (solid line) at 37 °C.

The increase in the GSH concentration up to 10 mM led to a faster release profile, with the release percentage increasing up to 42% in the first 30 min and raising 74% after 24 h (Figure 5). This different behavior, in accordance with the data of the destabilization studies, is ascribable to the breakage of the disulfide linkage. Prolonging the release time until 48 h, no significant enhancement of the release percentage was observed, probably for the strong drug to polymer interaction.

### 3.5. Cytotoxicity Studies

**Figure 6:**
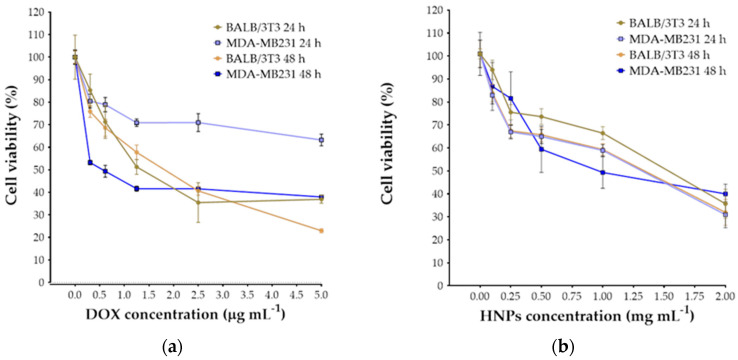
Determination of IC_50_ of free DOX (**a**) and self-assembling nanoparticles (HNPs) (**b**) for metastatic cancer (MDA-MB231) and healthy (BALB/3T3) cells after 24 and 48 h of cell culture.

The inhibition of cellular proliferation of DOX, HNPs and DOX@HNPs in a cancer cell line (MDA-MB231; human breast adenocarcinoma) and a normal cell line (BALB/3T3; mouse fibroblasts) was evaluated after 24 and 48 h using a CCK-8 assay. From these results, half-maximal inhibitory concentration (IC_50_) values of free drug and nanoparticle concentration were also calculated. Both cell lines showed a progressive decrease in cell survival in response to increasing concentrations of drugs and nanoparticles (Figure 6). For BALB/3T3 cells, IC_50_ values of DOX at 24 and 48 h were 1.25 and 1.5 μg mL^−1^, respectively. MDA-MB231 cells showed a significantly higher IC_50_ at 24 h (>10 μg mL^−1^), while at 48 h, IC_50_ was 0.62 μg mL^−1^. The high IC_50_ value of DOX at 24 h can be explained due to the drug resistance of the MDA cell line and the major role of efflux pumps, including *P*-glycoprotein [57]. Overall, BALB cells were significantly more sensitive to DOX compared to MDA cells. Similarly, the concentration of HNPs significantly affected the viability of both cancer and fibroblast cell lines. The IC_50_ of HNPs after 24 and 48 h incubation was >1 mg mL^−1^, except for the MDA cells at 48 h that showed an IC_50_ ≈ 1 mg mL^−1^. This finding suggested that HNPs could be easily uptake by MDA-MB231 cells due to the higher affinity for CD44 receptors present on the surface of the cells and resulting in a higher accumulation of nanoparticles in the intracellular space [26]. The proliferation inhibition studies for DOX@HNPs were carried out using an HNPs concentration of 0.1 mg mL^−1^ to ensure the effective formation of nanoparticles (as per CAC value of the conjugate) and negligible cytotoxic effects (as per IC_50_ value).

**Figure 7:**
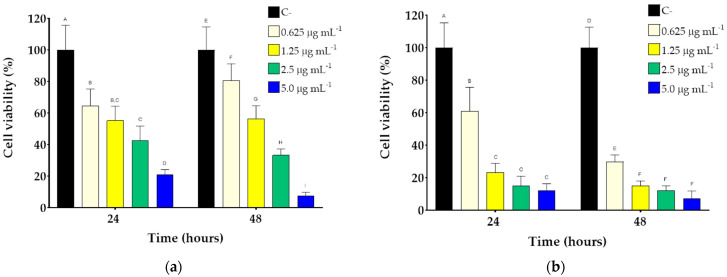
Cell viability of BALB/3T3 (**a**) and MDA-MB231 cells (**b**) treated with DOX@HNPs (drug concentration ranging from 0 to 5 µg/mL) after 24 and 48 h of culture. Within each group, different letters denote statistical differences for *p* < 0.05, *n* = 5.

The effects of DOX@HNPs on the inhibition of cell proliferation were evaluated using MDA-MB231 and BALB/3T3 cell lines (Figure 7). The increase in DOX concentrations led to a gradual decrease in the viability of both cancer and fibroblast cell lines. The cytotoxic effects of DOX@HNPs were significantly higher in MDA-MB231 cells than in fibroblasts when comparing the same DOX concentrations. After 24 h, the incubation of both cell lines with DOX@HNPs loaded with 0.625 μg mL^−1^ led to similar values of cell survival (64.6 ± 10.6% and 61.0 ± 14.6% for BALB and MDA, respectively). However, higher concentrations of DOX (1.25, 2.5, and 5 μg mL^−1^) were significantly more cytotoxic for MDA cells compared to BALB. Incubation of MDA cells with DOX@HNPs containing DOX concentrations of 1.25 μg mL^−1^ decreased cell viability to values close to 20% (23.3 ± 5.4%) after 24 h. However, BALB cell viability was not significantly more affected (55.3 ± 9.0%) at this concentration compared to DOX@HNPs 0.625 μg mL^−1^. The cytotoxicity of DOX-loaded HNPs was more pronounced after 48 h of incubation in contact with MDA cells. The lowest concentration of DOX@HNPs (DOX concentration of 0.625 μg mL^−1^) already showed a drastic decrease in MDA viability (29.8 ± 4.3%), compared to BALB cells (80.6 ± 10.6%). These results showed that DOX@HNPs are relatively safe in non-cancer cells such as BALB fibroblasts. Further information can be obtained by comparing the effect of free and encapsulated drugs on MDA-MB231. After 48 h of incubation, DOX@HNPs showed a marked decrease in cell viability compared to the free DOX (*p* < 0.05, *n* = 5) due to a more efficient cellular internalization in cancer cells overexpressing CD44. As previously reported, free DOX is internalized via passive diffusion, while DOX@HNPs enter the cells via endocytosis mediated by CD44 receptors and, furthermore, preventing efflux pump-associated drug activity [58,59,60]. Taken together, these results demonstrated the efficiency of the complexes in vitro and their effectiveness against cancer cells while avoiding the side effects of the free drug in normal cell lines.

### 3.6. Cellular Uptake

Cellular uptake of nanoparticles is one of the key factors that will dictate the efficacy of drug delivery in drug-NP complexes. The selective targeting of HNPs to CD44 (+) (MDA MB231) or (−) (BALB/3T3) cells was evaluated using fluorescent-labeled FITC-HNPs, obtained by covalent attachment of FITC on HAssHSA conjugate. The successful functionalization was confirmed by UV–Vis spectra, showing the appearance of the absorption peak of fluorescein moiety (Figure S1).

**Figure 8:**
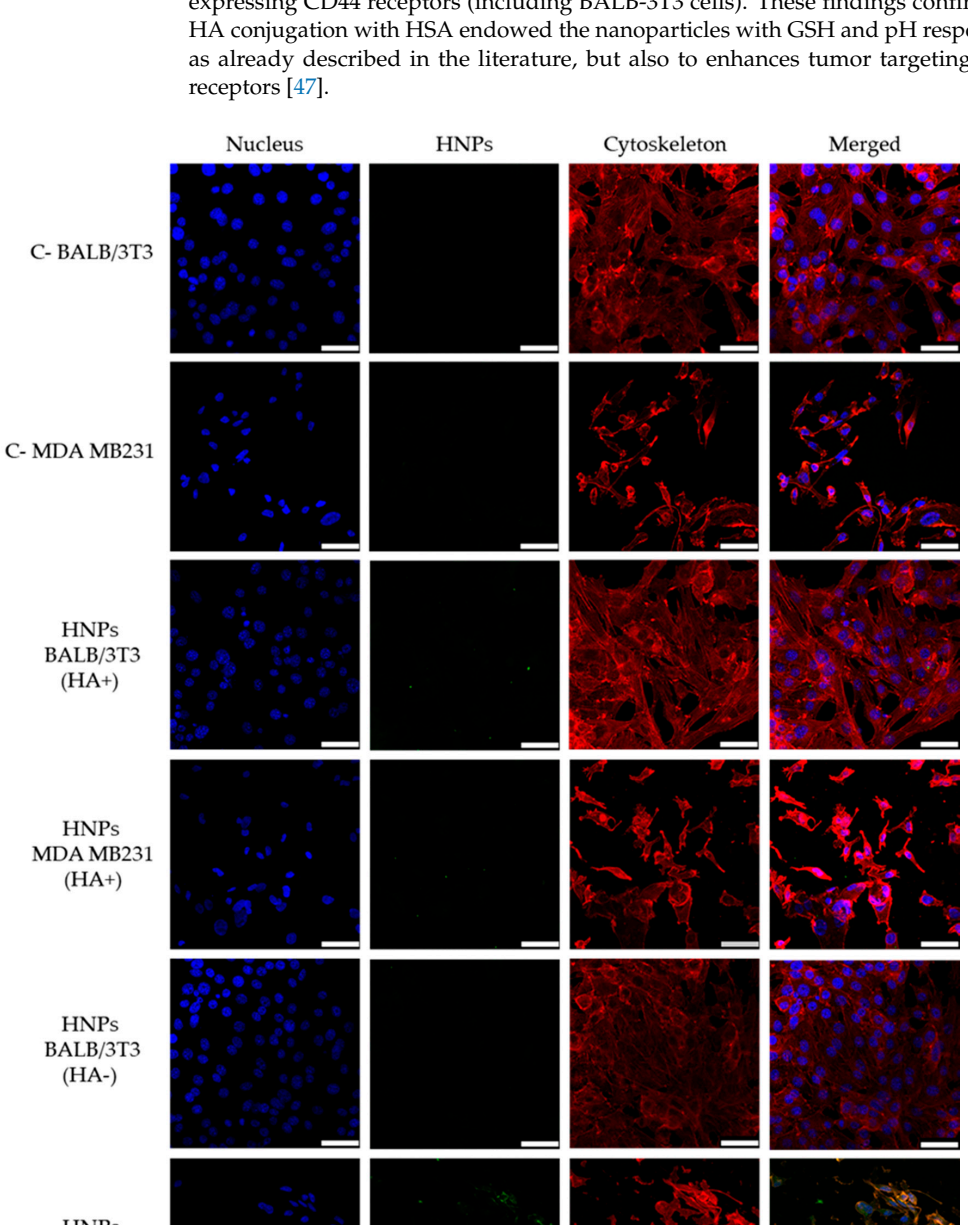
Representative confocal images of MDA-MB231 and BALB/3T3 cells exposed to HNPs (0.1 mg mL^−1^) for 24 h. CD44 receptors were blocked with free hyaluronic acid (HA) (10 equivalents of HNPs concentration) 1 h before treatment with HNPs. Green, blue, and red fluorescence correspond to fluorescein isothiocyanate (FITC)-HNPs, cell nuclei and cytoskeleton, respectively. Cells exposed to culture medium were used as the negative control. Scale bar: 50 µm.

In order to block CD44 receptors, cells were pretreated with free HA before incubation with FITC-HNPs, and then confocal analysis was used to visualize the intracellular presence of labeled nanoparticles. Three fluorescent channels were used to acquire confocal micrographs: cell nuclei were stained in blue using DAPI, cellular cytoskeleton (F-actin) was stained in red using Alexa Fluor 568 phalloidin, and FITC-HNPs were observed in green. As shown in Figure 8, FITC-HNPs exhibited an extensive cellular uptake and accumulated in the intracellular space when directly incubated with MDA-MB231 cells, as indicated by the green fluorescence observed in the areas surrounding cellular nuclei. In contrast, when cells were pre-incubated with free HA to block CD44 receptors, the cellular uptake of FITC-HNPs was markedly inhibited. Scattered extracellular aggregates of FITC-HNPs were observed after incubation with a concentrated (0.1 mg mL^−1^) nanoparticle dispersion, but the presence of intracellular nanoparticles was not evidenced in the microscopic examination. When CD44 negative cells (BALB/3T3) were incubated with FITC-HNPs, results showed a considerably lower accumulation of nanoparticles in the cytoplasmatic region, indicating that HNPs specifically target cells overexpressing CD44 receptors (Figure 8).

**Figure 9:**
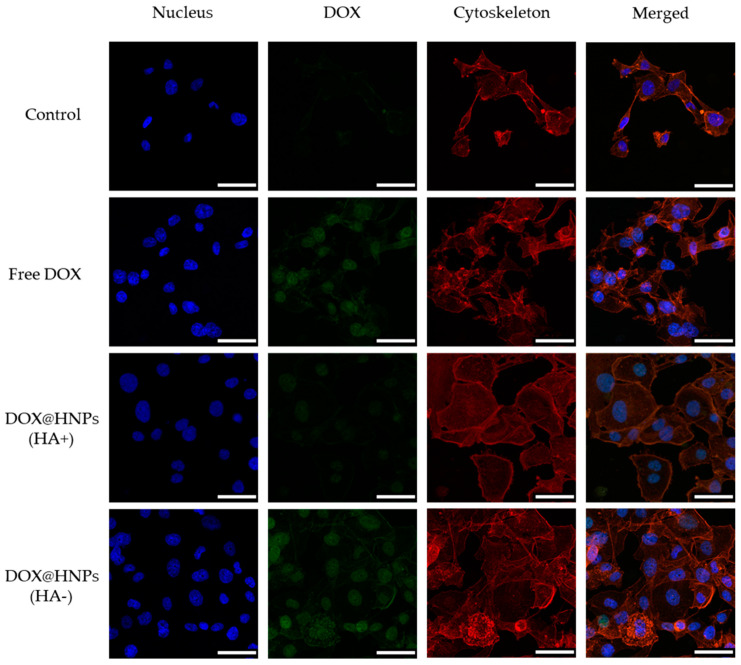
Representative confocal micrographs of MDA-MB231 cells incubated in culture medium (control), free DOX (0.8 μg mL^−1^), or DOX@HNPs (containing 0.8 μg mL^−1^ DOX) for 12 h. CD44 receptors were blocked (HA+) with free HA (10 equivalents of HNPs concentration) for 1 h before treatment with DOX@HNPs. Scale bar: 50 µm.

The internalization of DOX@HNPs was also evaluated using the self-fluorescence of DOX (Figure 9). MDA-MB231 cells incubated with free DOX showed an extensive accumulation of drugs in the nucleus due to the passive diffusion of the drug into the cells. The incubation of cells with DOX@HNPs for 12 h also showed the presence of intracellular DOX fluorescence in the nuclear regions. This result indicated that DOX molecules are released from HNPs after their internalization into the cell cytoplasmatic region. However, the incubation of cells with DOX@HNPs after CD44 receptors were blocked with HA showed a significant decrease in DOX concentration in the nuclear region of MDA-MB231 cells. The competition between free HA and HNPs to bind CD44 receptors indicate that HNPs exhibit a receptor-mediated cell internalization mechanism via endocytosis through the CD44 receptor [59]. These results are consistent with the cell proliferation inhibition studies and confirmed that HNPs had enhanced anticancer activity with CD44 receptor overexpressed cell lines (such as MDA-MB231 cells) rather than other cell lines under expressing CD44 receptors (including BALB-3T3 cells). These findings confirm that the HA conjugation with HSA endowed the nanoparticles with GSH and pH responsiveness, as already described in the literature, but also to enhances tumor targeting via CD44 receptors [47].

## 4. Conclusions

In this study, DOX-loaded micelle-like nanoparticles for targeted drug delivery were prepared by self-assembling of an HAssHSA conjugate obtained by covalent attachment of HSA to a cystamine-modified HA. The particle size (70 nm) endorsed the obtained nanoparticles with passive targeting properties via the EPR effect on tumor tissues. The composition of the amphiphilic conjugate enabled their active targeting for cancer cells via CD44 receptor interactions, a remarkable affinity for DOX, and stability at physiological pH. Furthermore, disulfide linkers can induce redox-triggered destabilization, thus modulating the drug release profiles. The efficacy of the proposed system was demonstrated by cell viability experiments, where DOX@HNPs showed a higher cytotoxic effect in MDA-MB231 cells than in healthy cells in comparison to free DOX. Finally, cellular uptake experiments confirmed the CD44-mediated internalization of nanoparticles in cancer cells. In conclusion, this study may enable a novel and safe strategy to prepare doxorubicin delivery systems for tumor targeting in cancer therapy. In vivo studies will be designed to further investigate the potential applications of the reported strategy.

## Data Availability

Not applicable.

## Supplementary Materials

The following are available online at https://www.mdpi.com/1999-4923/13/3/304/s1, Figure S1. UV–Vis spectra of FITC (4 μg mL^−1^) and FITC-HAssHSA (0.4 mg mL^−1^) dissolved in carbonate buffer (100 mM, pH 9.2).
