# Supplementary material for: Dual-Targeted Hyaluronic Acid/Albumin Micelle-Like Nanoparticles for the Vectorization of Doxorubicin
Source: Pharmaceutics. 2021 Feb 26;13(3):304. doi: 10.3390/pharmaceutics13030304 (PMC7996918; doi:10.3390/pharmaceutics13030304)
Supplement: Supplementary file 1 [file pharmaceutics-13-00304-s001.pdf]

pharmaceutics

Supplementary Materials: Dual-Targeted Hyaluronic Acid/Albumin Micelle-Like Nanoparticles for the Vectorization of Doxorubicin

Manuela Curcio, Luis Diaz-Gomez, Giuseppe Cirillo, Fiore Pasquale Nicoletta, Antonella Leggio and Francesca Iemma

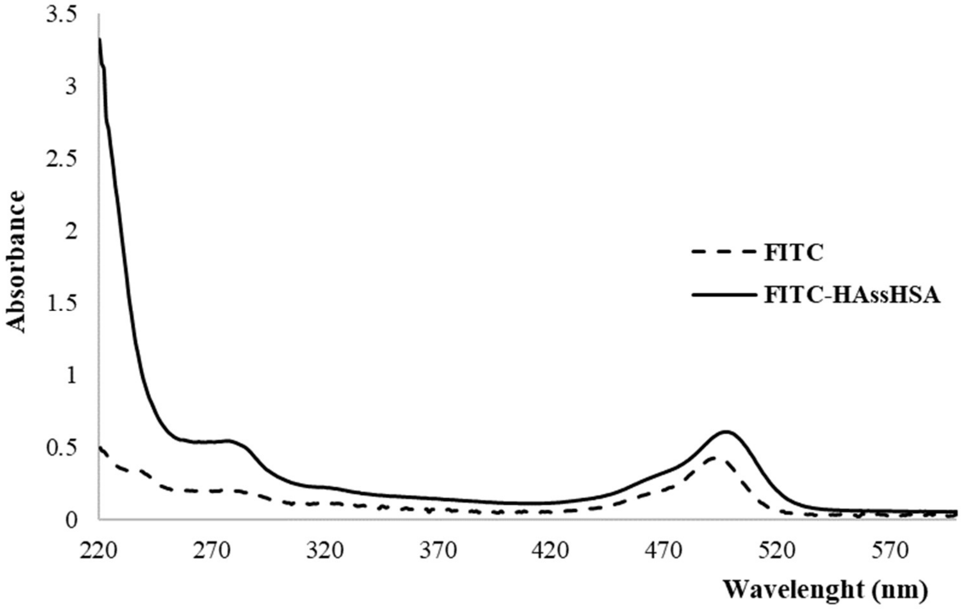

Figure S1. UV–vis spectra of FITC (4 μg mL^{-1}) and FITC-HAssHSA (0.4 mg mL^{-1}) dissolved in carbonate buffer (100 mM, pH 9.2).
